# Clinical efficacy of cyclosporin and natamycin for fungal keratitis

**DOI:** 10.12669/pjms.40.5.7777

**Published:** 2024

**Authors:** Chengwen Yang, Jingjing Cai, Qiuhong Wei, Huifang Lian, Lin An

**Affiliations:** 1 Chengwen Yang Department of Ophthalmology, Baoding NO.1 Central Hospital, Baoding 071000, Hebei, China; 2 Jingjing Cai Department of Ophthalmology, Baoding NO.1 Central Hospital, Baoding 071000, Hebei, China; 3 Qiuhong Wei Department of Ophthalmology, Baoding NO.1 Central Hospital, Baoding 071000, Hebei, China; 4 Huifang Lian Department of Ophthalmology, Baoding NO.1 Central Hospital, Baoding 071000, Hebei, China; 5 Lin An Department of Ophthalmology, Baoding NO.1 Central Hospital, Baoding 071000, Hebei, China

**Keywords:** Cyclosporin, natamycin, Fungal keratitis, Clinical efficacy

## Abstract

**Objectives:**

To investigate the clinical efficacy of cyclosporin (CYSP) and natamycin (NAT) as a combination therapy in patients with fungal keratitis.

**Methods:**

This is a retrospective study. A total of 64 patients (64 eyes) with fungal keratitis treated by Baoding No.1 Central Hospital between December 2018 and May 2022 according to their treatment methods were divided into a monotherapy (MT) group receiving NAT eye drops solely and a combination therapy (CT) group given CYSP eye drops in addition to the exact treatment provided for the MT group. The clinical responses, visual acuity changes, severity of eye symptoms, and adverse reactions were compared between the two groups.

**Results:**

At two and four weeks post-treatment, the CT group had an overall response rate (ORR) significantly higher than that of the MT group (*P*< 0.05, respectively); both groups showed improved visual acuity and eye symptoms compared with the pre-treatment condition, and these improvements were more pronounced in the CT group (*P* < 0.05, respectively). Compared with the MT group, the CT group experienced a significantly shorter duration of eye symptoms (*P* < 0.05). The adverse reaction rate(ARR) was 9.38% in the CT group and 6.25% in the MT group, and the difference was not significant (*P* > 0.05).

**Conclusion:**

Using CYSP and NAT as a combination therapy for fungal keratitis can substantially heighten the therapeutic effects, promote visual acuity recovery, and induce rapid remission of eye symptoms without increasing the risk of adverse reactions.

## INTRODUCTION

Fungal keratitis is a common infectious keratitis disease mainly caused by pathogenic bacteria such as Fusarium and Eurotium.^1^-^2^ Characterized by the slow growth of fungi and the infective process for up to several months, fungal keratitis can lead to corneal ulcers, vision impairment, and even blindness.^3^-^4^ Therefore, clinical treatment should focus on the early control of fungal keratitis to prevent progression and deterioration. To date, the clinical treatment of fungal keratitis mainly depends on antifungal drugs and surgery because specific remedies have not yet been developed.^5^ Compared with surgical treatment, antifungal drugs have gained wider traction and application because of their accessibility and safety profiles.^6^ Natamycin(NAT) is a commonly used antifungal medication for patients with fungal keratitis to combat the pathogenic fungi and alleviate eye symptoms in clinical settings. However, this type of infection is typically difficult to treat. Most severely infected patients do not respond well to the use of a single antibacterial agent.^7^ To overcome this limitation, the present study introduced a combination therapy composed of NAT and cyclosporin(CYSP) for patients with fungal keratitis.

## METHODS

This is a retrospective study included 64 patients with fungal keratitis who visited Baoding No.1 Central Hospital from December 2018 to May 2022. The subjects according to their treatment methods were divided a monotherapy(MT) group and a combination therapy(CT) group(n= 32 in each group, 64 eyes in total). No significant differences were found between the two groups in demographic and clinical data (*P*> 0.05, respectively) (Table-I).

**Table-I:** Demographic and clinical data

Group	Sex (n)		Age (yr)		Disease course (mth)		Infection severity		
			
	Male	Female	Range	Mean age	Range	Mean disease course	Mild	Moderate	Severe
CT group (n = 32)	17	15	22-42	32.54±2.41	1-5	2.87±0.54	6	15	11
MT group (n = 32)	18	14	21-44	31.96±3.16	1-16	2.95±0.67	5	16	11
*χ^2^/t*	0.063			0.826		0.526	0.123		
*P*	0.802			0.412		0.601	0.940		

### Ethical Approval

The study was approved by the Institutional Ethics Committee of Baoding NO.1 Central Hospital (No.:[2022]052; November 3, 2022), and written informed consent was obtained from all participants.

Inclusion criteria:

- Patients who met diagnosis of fungal keratitis.^8^
- Patients in conformity with the diagnostic criteria published by the WHO.
- Patients were confirmed by a smear test, corneal scraping, and confocal microscopy.
- Patients who were informed consent to the present study.

### Exclusion criteria:

- Patients who were known allergy to NAT and/or CYSP.
- Patients with concurrent ocular conditions.
- Patients who were pregnant or breast-feeding women.
- Patients with comorbid severe liver and/or kidney dysfunction.
- Patients with a systemic or severe local infection.
- Patients who had a noxious ocular disease caused by diabetes or other conditions.
- Patients with immunological or mental disorders.
- Patients with a history of eye surgery.
- Patients who were failure to fully cooperate during the study.

The MT group was treated with the NAT eye drops (G.Y.Zh.Z. H20083293) produced by the North China Pharmaceutical Company via the ophthalmic route. For patients with mild to moderate infection, 1-2 drops were administered every hour; for severe cases, NAT was given three doses every two hours, 1-2 drops per dose, for 2-4 weeks.

The CT group used the exact NAT eye drops in combination with the CYSP eye drops (G.Y.Zh.Z. H20070106) provided by the same manufacturer. The dosage and frequency varied according to infection severity: mild and moderate cases: four doses daily, 1-2 drops per dose; severe cases: six doses daily, 1-2 drops per dose. The two antifungal drugs were given at least 10 minutes apart from each other during each administration, for 2-4 weeks.

### Outcome measures

According to the criteria set out by Zeng et al., clinical responses were evaluated at two and four weeks post-treatment.^9^ Complete response(CR): the disappearance of hypopyon and relevant clinical symptoms, full recovery of visual acuity, complete healing of corneal ulcers, and negative fluorescein staining; partial response(PR): evident improvements in hypopyon and relevant clinical symptoms, partial recovery of visual acuity, significant healing of corneal ulcers, and negative fluorescein staining; no response(NR): no observable improvements in hypopyon, relevant clinical symptoms, visual acuity, or corneal ulcers, and positive fluorescein staining. Overall response rate(ORR)= (CR cases + PR cases) / total cases × 100.0%.

Visual acuity and eye symptoms Visual acuity was measured in decimals pre- and at two and four weeks post-treatment. Eye symptoms were assessed using a fungal keratitis severity scale(FKSS) independently developed by Baoding No.1 Central Hospital (Cronbach’s α =0.83; reliability =0.82; validity =0.81). The nine-point FKSS has three items: anterior chamber reactions, lesion size, and lesion depth, and each item is scored from one to three, with a higher score indicative of more severe eye symptoms and worse recovery.

Time to complete remission of eye symptoms were observed and documented, such as hypopyon, corneal ulcers, sensitivity to light and tearing, and foreign body sensations.

### Adverse reactions

These were compared between the two groups. The two groups of patients were followed up for three months.

### Statistical analysis

Data analysis was performed using the SPSS25.0 software program. Enumeration data were expressed in the form of “n(%)”, and comparisons were analyzed with the *χ^2^* test. Comparisons of ranked data were examined by the Wilcoxon Rank Sum Test. Measurement data were represented by “*χ̅*±*S*”; intragroup comparisons were made by the paired t-test and intergroup comparisons by the independent-samples t-test. The confidence interval was 95%. P-values less than 0.05 (*P*< 0.05) were considered statistically significant.

## RESULTS

The ORR was significantly higher in the CT group than in the MT group at both two and four weeks post-treatment (*P* < 0.05, respectively). Table-II & III. Before treatment, the two groups had no significant differences in visual acuity and the FKSS score for eye symptoms (*P* > 0.05, respectively). At two and four weeks post-treatment, both groups exhibited improvements in visual acuity and the FKSS score; notably, the CT group made a greater improvement in visual acuity and had a significantly lower FKSS score compared with the MT group (*P* < 0.05, respectively). Table-IV. Eye symptoms disappeared significantly faster in the CT group compared with the MT group (all *P* < 0.05). Table-V.

**Table-II:** Clinical responses at 2 weeks post-treatment [n(%)].

Group	CR	PR	NR	ORR
CT group (n = 32)	15(46.88)	11(34.38)	6(18.75)	26(81.25)
MT group (n = 32)	8(25.00)	9(28.13)	15(46.88)	17(53.13)
*Z/χ^2^*	6.188			5.741
*P*	0.045			0.017

**Table-III:** Clinical responses at 4 weeks post-treatment [n(%)].

Group	CR	PR	NR	ORR
CT group (n = 32)	18(56.25)	12(37.50)	2(6.25)	30(93.75)
MT group (n = 32)	10(31.25)	14(43.75)	8(25.00)	24(75.00)
*Z/χ^2^*	2.366			4.267
*P*	0.018			0.039

**Table-IV:** Visual acuity and FKSS score (*χ̅*±*S*)

Group	Visual acuity			FKSS score		

	Pre-treatment	At 2 weeks post-treatment	At 4 weeks post-treatment	Pre-treatment	At 2 weeks post-treatment	At 4 weeks post-treatment
CT group (n = 32)	0.13±0.02	0.30±0.04^a^	0.38±0.12^a^	4.08±1.26	2.03±0.1^a^2	1.02±0.35^a^
MT group (n = 32)	0.14±0.03	0.18±0.05^a^	0.28±0.11^a^	3.97±1.06	2.85±0.35^a^	2.56±0.71^a^
*t*	1.569	10.601	3.475	0.378	12.537	11.005
*P*	0.122	<0.001	<0.001	0.707	<0.001	<0.001

***Note:***

a P < 0.05 when comparing the pre- and post-treatment visual acuity and FKSS scores within each group.

**Table-V:** Time to complete remission of eye symptoms [d, (*χ̅*±*S*)].

Group	Hypopyon	Corneal ulcers	Sensitivity to light and tearing	Foreign body sensations
CT group (n = 32)	13.65±2.68	14.20±2.51	16.57±3.87	14.26±2.79
MT group (n = 32)	16.24±3.52	18.98±3.26	21.64±3.43	19.64±3.36
*t*	3.312	6.572	5.546	6.969
*P*	0.002	<0.001	<0.001	<0.001

Adverse reactions during the treatment course were mainly redness and swelling (CT group: n= 2; MT group: n= 1), and itching (CT group: n = 1; MT group: n = 1). The total adverse reaction rate (ARR) was 9.38% in the CT group and 6.25% in the MT group, and the difference was not significant (*χ^2^*=0.217, *P* > 0.05). All adverse reactions were mild, and thus no special treatment was needed.

## DISCUSSION

Significant clinical efficacy was observed in the CT group simultaneously treated with NAT and CYSP. Comparing the CT group with the MT group, eye symptoms disappeared more quickly, and visual acuity was improved to a higher level. CYSP eye drops benefit patients with fungal keratitis through enhancement of corneal immune protection, which increases the bioavailability and improves the overall efficacy. The active immunosuppressant CYSP is essentially an 11-amino acid cyclic peptide derived from fungal metabolites. It is believed that CYSP serves as a selective inhibitor of T cell subsets (including T helper cells and B cells) to suppress cell-mediated immunity.^10^ Moreover, CYSP is considered destructive to the growth of Fusarium solani and Aspergillus fumigatus because of its fungistatic activity as a fungal metabolite, which achieves suppression of competitive fungal growth through inhibition of toxins.

Fungal keratitis is a common clinical eye infection predominantly caused by Fusarium and Eurotium.^11^-^12^ Patients with fungal keratitis may develop inflammatory responses in the cornea and anterior chamber, which can give rise to corneal ulcers and perforation, induce hypopyon, and result in blindness_._^13^ Clinically, antifungal drugs have long been the standard of care for fungal keratitis.^14^-^15^ However, the increasingly widespread use of antibacterial agents makes drug resistance an emerging challenge to ophthalmologists. This underpins a need to improve the clinical efficacy for fungal keratitis through the scientific use of antibacterial drugs.^16^-^17^

NAT eye drops are frequently used in the clinical treatment of fungal keratitis. This antifungal agent fights off a wide range of pathogenic filamentous fungi and yeast through direct contact with the corneal ulcer surface.^18^,^19^ Nevertheless, data from several studies suggest that patients with moderate to severe fungal keratitis respond poorly to NAT as a monotherapy.^20^,^21^ The mechanism underlying its antifungal activity is closely associated with a special component known as a tetraene polyene antibiotic. This substance seems to bind to the sterol moiety of the fungal cell membrane and thereby form a polyenesterol complex that alters the permeability of the membrane and promotes fungal cell death. Previous research has established that NAT eye drops deliver superior therapeutic effects by killing the major pathogenic organisms - Fusarium and Eurotium - on fungal cell membranes.^22^

Evidence also suggests that NAT achieves significant, long-lasting antifungal effects by binding to the necrotic substances in lesions.^23^ Despite all that, NAT, owing to its poor penetration ability, demonstrates relatively low bioavailability and an inferior efficacy profile in treating a deep corneal infection.^24^ Fungal keratitis-induced corneal damage entails a risk of immunomodulatory disorders and diminished antimicrobial activity. This is likely to result in further aggravation of immunopathological damage. NAT eye drops, when combined with CYSP, are discovered to exert markedly improved therapeutic effects without triggering any significant adverse reactions.^25^ This finding is similar to the results reported in this study. CYSP belongs to a family of neutral, lipophilic, cyclic oligopeptides that pass easily through the corneal epithelium and accumulate in the corneal stroma.

This makes it a better penetrant than NAT to reach an effective therapeutic concentration level in less time and achieve desirable therapeutic effects. Therefore, using NAT and CYSP as a combination therapy may offer complementary and synergistic benefits and improve the clinical responses of patients with fungal keratitis. Furthermore, the use of CYSP eye drops is reported to be associated with a reduced recurrence rate of fungal keratitis.^26^ A probable explanation is that as an immunosuppressive agent, CYSP can expedite the healing of corneal ulcers and prevent recurrent inflammation by topical application. In clinical practice, antifungal eye drops should be administered after local debridement to kill the pathogenic fungi in deeper layers and achieve better treatment outcomes.

### Limitations of the study

However, the small sample size does not allow analysis and comparison of clinical efficacy across patients with varying degrees of fungal keratitis. A wider spectrum of further in-depth studies with a larger sample size are needed to investigate and compare the therapeutic effects on patients with mild, moderate, and severe fungal keratitis, validate the application value of this combination therapy for the affected population and support the findings of the present study.

## CONCLUSION

The combination therapy of CYSP and NAT, with improved efficacy and preferable safety profiles, appears to substantially improve the visual acuity and timely relieve the eye symptoms of patients with fungal keratitis.

### Authors’ Contributions:

**CY** and **JC:** Carried out the studies, participated in collecting data, and drafted the manuscript, are responsible and accountable for the accuracy and integrity of the work.

**QW** and **HL:** Performed the statistical analysis and participated in its design.

**LA:** Participated in acquisition, analysis, or interpretation of data and draft of the manuscript.

All authors read and approved the final manuscript.
